# Complementing ODE-Based System Analysis Using Boolean Networks Derived from an Euler-Like Transformation

**DOI:** 10.1371/journal.pone.0140954

**Published:** 2015-10-23

**Authors:** Claudia Stötzel, Susanna Röblitz, Heike Siebert

**Affiliations:** 1 Mathematics for Life and Materials Sciences, Zuse Institute Berlin, Berlin, Germany; 2 Dep. of Mathematics and Computer Science, Freie Universität Berlin, Berlin, Germany; King’s College London, UNITED KINGDOM

## Abstract

In this paper, we present a systematic transition scheme for a large class of ordinary differential equations (ODEs) into Boolean networks. Our transition scheme can be applied to any system of ODEs whose right hand sides can be written as sums and products of monotone functions. It performs an Euler-like step which uses the signs of the right hand sides to obtain the Boolean update functions for every variable of the corresponding discrete model. The discrete model can, on one hand, be considered as another representation of the biological system or, alternatively, it can be used to further the analysis of the original ODE model. Since the generic transformation method does not guarantee any property conservation, a subsequent validation step is required. Depending on the purpose of the model this step can be based on experimental data or ODE simulations and characteristics. Analysis of the resulting Boolean model, both on its own and in comparison with the ODE model, then allows to investigate system properties not accessible in a purely continuous setting. The method is exemplarily applied to a previously published model of the bovine estrous cycle, which leads to new insights regarding the regulation among the components, and also indicates strongly that the system is tailored to generate stable oscillations.

## Introduction

When modeling biological phenomena, different levels of abstraction can be used to capture the mechanisms of the underlying system [1]. Ordinary differential equations (ODEs) are well suited to describe interactions based on concentration changes and allow for detailed predictions. However, due to the complexity of the models, it is often not possible to perform a global analysis of dynamical properties. Additionally, the model behavior strongly depends on the current parameter values such as rate constants, and the quantitative data necessary to identify them is often lacking [2]. In this case, qualitative modeling, e.g., using Boolean formalisms, constitutes a fruitful alternative. Herein, the system is described on a higher level of abstraction strongly based on the network structure and a logical description of interaction mechanisms. While the coarseness of such models does not allow for high resolution of model output, it does permit a much more global analysis of a system due to a finite state space and has shown to be capable of uncovering fundamental characteristics and functionalities [3].

Common to all modeling formalisms is the fact that a model always constitutes a simplification of reality, focusing on particular aspects of interest and neglecting details either by design or simply due to lack of knowledge. Utilizing different modeling approaches in parallel may therefore help to produce a more rounded picture of a system’s capabilities and to highlight modeling artifacts (see e.g. [4–6]).

We aim at exploiting the strengths and weaknesses of ODE and logical modeling by using them in concert. Taking the opposite direction as [7], we start from an ODE system and propose a method to derive a Boolean model offering a new point of view on the system without having to repeat an explicit modeling process to obtain the more abstract model. The simplicity of the Boolean representation allows for a much more comprehensive analysis of its dynamics to complement the more local results obtained from the ODE system. A global attractor analysis as often infeasible for ODE models, e.g., allows to understand capabilities of the system in terms of stable behavior and to uncover decision processes determining long-term dynamics. In application, such insights are of particular interest when analyzing differentiation or multi-stable response systems [8, 9]. The easy adaption of such models to knock-out/in scenarios facilitates extensive perturbation studies [10]. In addition, the coarser representation allows for easier identification of structural properties that are fundamental for specific dynamical effects, e.g., based on a feedback circuit analysis [11].

The main result of our paper is a new, systematic and simple method to obtain a Boolean from an ODE model for the purpose of enhancing system analysis. Existing approaches of relating continuous and logical models can roughly be divided according to two different goals. In one research line, the interest lies in capturing the way system properties are conserved when discretizing continuous models in a rigorous mathematical way. Here, good results can be achieved when focusing on small classes of ODE models where the translation into a discrete setting is rather straightforward [12]. In [13], a method is proposed to map continuous models of biochemical control networks, in which the control functions are continuous homologues of logical control functions, to discrete models. For a restricted subclass of dynamical systems, a correspondence regarding certain qualitative dynamical features has been proven. Later, the authors in [14] analyzed transitions of dynamics in randomly constructed ODE analogues of random Boolean networks.

In other works, the emphasis is more on the application side, aiming at deriving discrete models useful for system analysis. As in [15], such methods are often only described closely intertwined with a case study. This leads to an individual solution that depends on many regulation-based distinctions and steady state assumptions. In [15], the transformation from ODEs to Boolean networks is done in a controlled way, keeping the dynamical properties in mind. They find that the transition is possible for differential equations which have monotonic sigmoidal functions with distinct upper and lower asymptotes. Finally, the author show that the Boolean model reproduces the results of the initial ODE model.

In this paper, we do not focus on conservation of desired dynamical properties during the translation process. Instead, we propose a systematic and thus easily accessible, step-wise procedure of translation with subsequent validation and analysis suitable for a large class of ODE models interesting for application. Discretization of the continuous system is based on the Euler method, a well-known tool for the numerical approximation of continuous dynamical systems (see e.g [16] for a general reference and [17], Chapter 8, for a related application). We only use the structure and monotonicity behavior of the right hand side functions of the ODEs for directly deriving the discrete model. Since in general conservation of specific dynamical properties cannot be predicted a priori, we proceed with a validation step to check if at least the properties of interest are preserved. This allows for the construction of feasible models without the prerequisite of fulfilling specific mathematical assumptions in advance. Nevertheless, the corresponding Boolean model preserves the qualitative biological information comprised in the ODE model, namely the underlying network structure and the processing logic of regulatory effects, and thus includes the information that would be incorporated in a traditional bottom-up modeling procedure for Boolean networks.

The validation step ensures that the derived Boolean model satisfies the given requirements in terms of property conservation. It can be conducted utilizing Boolean-like biological data, aiming at a model capturing all important biological observations. A different possibility is to validate the Boolean with respect to the ODE model by generating data from the simulation of the latter for the purpose of validation. In particular, this might be of interest if data on the real system is lacking or if analysis of the Boolean model aims at providing pointers for a more targeted analysis of the ODE model. When the system functionality of interest is governed by qualitative regulation processes, as often the case, e.g., in differentiation, bistable switching or signal processing, validation is very likely to succeed. Note that, even if validation would fail, we always gain information on the system since the discretization procedure pinpoints processes whose functionality crucially depends on certain quantitative processes. Analysis of the Boolean model then concludes the procedure. As mentioned, the focus can either lie on gathering new insights into the modeled system or aiming at a more comprehensive analysis of the ODE model.

In the first part of this paper, we present the general approach to deriving a Boolean from a given ODE model. Then, since the validation step depends on the availability of data as well as on the intended purpose of the model, we describe some validation possibilities often of use in application. Similarly, we shortly discuss possible analysis approaches for the discrete model to round off the description of the method. To illustrate the procedure, we apply the approach to a medium-sized model of the bovine estrous cycle, named *BovCycle*, that has been presented in [18, 19]. Utilizing the generated Boolean model, we investigate stability, prerequisites for certain observed component activities, and the impact of self-regulatory effects. In contrast to steady states, proving conservation of cyclic attractors in a purely mathematical setting is notoriously difficult. The proposed validation method demonstrates how to check the conservation of oscillatory behavior and is suitable for both transient trajectories and attractors. It nicely illustrates the potential of our more pragmatic approach in application.

## Results

In this section, we present our procedure for deriving a discrete model from an ODE model. We formulate assumptions for the ODE model (Step 0), and perform an Euler-like discretization (Step 1) which leads to a Boolean model. We then propose strategies for validation (Step 2) and analysis (Step 3) of the discrete model according to the given specifics.

### Proposed Method for the Discretization of an ODE Model

In the following, we describe the four steps of our discretization scheme.

#### Step 0: Preliminaries and Assumptions

As starting point, we consider an autonomous system of ODEs in the form

$$\begin{array}{c} y^{\prime }\left(t\right)=f\left(y,p\right),\;y\left(0\right)=y_{0} \end{array}$$

with time variable $t\in {\mathbb{R}}_{\geq 0}$, state vector $y(t)={\left(y_{1}(t),\ldots ,y_{n}(t)\right)}^{T}\in {\mathbb{R}}^{n}$, model parameters $p\in {\mathbb{R}}^{q}$, and right hand side $f:{\mathbb{R}}^{n\times q}\mapsto {\mathbb{R}}^{n}$. For simplicity of notation, the parameter vector *p* will be omitted in the following.

Throughout the paper, we assume that all continuous variables *y*_*i*_(*t*) are normalized to the interval [0, 1]. This can easily be achieved for every given ODE system by rescaling of model variables and parameters. Accordingly, initial values *y*(0) are also non-negative and bounded by one.

In addition, we assume that the right hand side functions *f*(*y*) = (*f*_1_(*y*), …, *f*_*n*_(*y*))^*T*^ consist only of sums and products of *monotone* functions *F*_*i*,*j*_(*y*_*j*_),

$$\begin{array}{c} y_{i}^{\prime }\left(t\right)=f_{i}\left(F_{i,1}\left(y_{1}\right),\ldots ,F_{i,n}\left(y_{n}\right)\right),\;\;\;i=1,\ldots ,n, \end{array}$$ (1)

whereby each monotone function *F*_*i*,*j*_(*y*_*j*_) only takes values in [0, 1], which can be achieved by appropriate shifting and scaling. An example for such a function *F*_*i*,*j*_(*y*_*j*_) is the increasing Hill function

$$\begin{array}{c} H^{+}\left(y_{j},T,r\right)=\frac{y_{j}^{r}}{y_{j}^{r}+T^{r}} \end{array}$$

with model parameters *T* and *r*. An example for the combination of two monotone functions is the product of two species

$$\begin{array}{c} F_{i,j}\left(y_{j}\right)\cdot F_{i,k}\left(y_{k}\right)=y_{j}\cdot y_{k} \end{array}$$

as it typically occurs in the modeling of a bimolecular reaction. Monotonicity is not a strong requirement in the context of modeling biological systems because classical mass action kinetics as well as Michaelis-Menten kinetics, Goldbeter-Koshland kinetics or Hill kinetics all fall into the class of monotone functions.

#### Step 1: Translating the ODE System into a Logical Model by an Euler-like Discretization

In the discrete counterpart of the ODE model, each real-valued variable *y*_*i*_(*t*), *i* = 1, …, *n*, is represented by a corresponding discrete variable *x*_*i*_. In the following, we will focus on deriving a Boolean network from the ODE model, i.e., each variable *x*_*i*_ takes values in the set {0, 1}, yielding the state space {0, 1}^*n*^.

For this purpose, the update function *g* : {0, 1}^*n*^ → {0, 1}^*n*^ of the Boolean model needs to be constructed. The goal is to capture the regulatory effects given by the ODE model, expressing them in an explicit form describing the state transitions directly rather than the implicit form of the ODE system. Clearly, the coarser nature of the logical model will emphasize certain effects while smoothing out others. However, the qualitative behavior of the ODE model should be preserved.

In our method, the expression “Euler-like step” refers to its similarity with the explicit Euler method for the numerical integration of ODEs. The idea is to evaluate the difference between a current state *s* of the ODE system and the state obtained by one Euler step, i.e., the state obtained by approximating the trajectory starting in *s* by the tangent in *s* and following it for a step of given length. In the discrete setting, we will discretize the impact of the regulators and only evaluate whether the tangent in *s* indicates a value increase, decrease or no value change at all.

To this end, we replace all continuous variables *y*_1_, …, *y*_*n*_ in the right hand side functions *f*_*i*_(*y*) by their discrete counterparts *x*_1_, …, *x*_*n*_. Thereby, every monotonically increasing function *F*_*i*,*j*_(*y*_*j*_) is mapped to *x*_*j*_, and every monotonically decreasing function *F*_*i*,*j*_(*y*_*j*_) is mapped to (1 − *x*_*j*_). We thus define an operator

$$\begin{array}{c} T\left(F\right):=\{\begin{array}{cc} Id & ,\;\text{if}\;F\;\text{is}\;\text{monotonically}\;\text{increasing},\\ 1-Id & ,\;\text{if}\;F\;\text{is}\;\text{monotonically}\;\text{decreasing}, \end{array} \end{array}$$

and map the right hand sides *f*_*i*_(*y*_1_, …, *y*_*n*_) in Eq (1) to discrete counterparts *h*_*i*_(*x*_1_, …, *x*_*n*_),

$$\begin{array}{c} f_{i}\left(F_{i,1}\left(y_{1}\right),\ldots ,F_{i,n}\left(y_{n}\right)\right)\mapsto f_{i}\left(\underset{x_{1}\;\text{or}\;1-x_{1}}{\underset{︸}{TF_{i,1}\left(x_{1}\right)}},\ldots ,\underset{x_{n}\;\text{or}\;1-x_{n}}{\underset{︸}{TF_{i,n}\left(x_{n}\right)}}\right)=:h_{i}\left(x_{1},\ldots ,x_{n}\right), \end{array}$$

for all *i* = 1, …, *n*.

Note that the values of the thus defined functions $h_{i}:{\left\{0,1\right\}}^{n}\to \mathbb{R}$ depend not only on the values of the Boolean variables *x*_1_, …, *x*_*n*_, but also on the model parameters *p*. Depending on these values, *h*_*i*_(*x*) = *h*_*i*_(*x*_1_, …, *x*_*n*_) can either be negative, positive, or equal to zero. The sign of *h*_*i*_(*x*) then determines the update rule for the Boolean variables. The update function *g* is defined as *g* : {0, 1}^*n*^ → {0, 1}^*n*^ with

$$\begin{array}{c} g_{i}:{\left\{0,1\right\}}^{n}\to \left\{0,1\right\}:x\mapsto g_{i}\left(x\right):=\{\begin{array}{cc} 1 & ,\;\text{if}\;sgn(h_{i}\left(x\right))>0\\ x_{i} & ,\;\text{if}\;sgn(h_{i}\left(x\right))=0\\ 0 & ,\;\text{if}\;sgn(h_{i}\left(x\right))<0 \end{array},\;\;\;i=1,\ldots ,n \end{array}$$ (2)

This can be interpreted as an Euler discretization step yielding the successor state *x* ^*k* + 1^ for a current state *x* ^*k*^ in the form

$$\begin{array}{c} x_{i}^{k+1}=g_{i}\left(x^{k}\right)=x_{i}^{k}\dot{+}sgn\left(h_{i}\left(x_{1}^{k},\ldots ,x_{n}^{k}\right)\right), \end{array}$$

where $\dot{+}$ denotes the operation $0\dot{+}(-1)=0$ and $1\dot{+}1=1$ and the usual addition in all other cases.

**Remark:** Step 1 can be applied in a generic manner to all ODE models satisfying the given conditions and results in a fully specified Boolean model. The following two steps, validation and analysis of the Boolean model, are highly dependent on the available data and the intended purpose of the model. Consequently, we aim at illustrating the two steps by discussing possible goals and well-established techniques.

#### Step 2: Validation of the Boolean Model

As mentioned before, the Boolean model can on one hand be considered as another representation of the biological system or, alternatively, it can be used to complement the analysis of the original ODE model. Depending on the purpose, two different data sources can be used to validate the model. In the first case, one should use available experimental data from the biological system of interest. In this case, the Boolean model, regardless of its construction, is in some sense uncoupled from the ODE model. The validation proceeds just as in the case of a model directly constructed from biological data.

In the second case, one should check whether the Boolean model conserves the behavior of the corresponding ODE model, or at least specific aspects of the behavior deemed to be of central interest. Note that we generally cannot ensure such a conservation based on the formal relation between the two models, as is possible to mathematically prove under more rigorous conditions [13, 14]. In this case simulated data from the ODE can be used for the validation procedures. Note that in general one would use simulations that are a good fit to the experimental observations underlying the ODE model, so indirectly this amounts to a validation according to the biological observations as well.

For the validation we concentrate on the dynamical behavior of the model, i.e., its state transition graph. Here, we consider the asynchronous update strategy to derive the state transitions (see Materials and Methods). This update strategy can be viewed as an over-approximation of the system behavior since it takes all possibilities for time delay orderings of regulatory processes into account. The result is a non-deterministic representation, which has been shown to include highly realistic trajectories [17].

We now shortly describe two validation strategies particularly useful in application. For both strategies, either data originating from lab experiments or from the simulation of the ODE model can be utilized.

One common approach exploits time series data. Within the discrete modeling formalism, we can utilize time series measurements as well as more qualitative observations, e.g., a sequence of activation events. Whenever a time series with quantitative measurements is available, we first need to discretize it. For a discussion of suitable discretization methods see [20, 21] and references therein. A discrete time series can be seen as a sequence of states that the system needs to visit in the given order. Additional constraints can be formulated to pose more strict demands on the trajectory, e.g., that component values are not allowed to oscillate between measurements or that the trajectory does not exceed a certain length. To validate the model we thus check whether the model is capable of generating such a trajectory. This can be done very efficiently using formal verification methods (see e.g. [22]).

Another type of data that is readily exploited for validation of discrete models is data from knockout or knock-in experiments. Here, the Boolean model is easily adapted to the perturbed system by fixing the update function of the over- or under-expressed components to the corresponding value [3]. The altered and the non-perturbed model dynamics can then be compared with the experimental observations, e.g., with respect to steady state behavior. Similarly, differentiation capabilities of a system, e.g., represented by bi-stability, can be verified by checking for corresponding attractors of the model.

If validation fails, the next step is to pinpoint the problematic aspects of the model (see e.g. [3, 22] for possible approaches). Comparison with the underlying ODE model can help to identify mechanisms dependent on gradual responses, e.g., concentration gradient dependent processes, that can not be easily captured in a Boolean setting.

Correcting the model, e.g., by adapting the update function of a specific component or adding components to achieve a finer resolution, can then yield a model capable of generating the observed behavior that can be used for further analysis of the biological system. Naturally, the necessary changes need to be analyzed in light of their biological interpretation. Usually, many different changes are possible, and adapting the model can be seen as hypothesis generation for not fully understood mechanisms that need to be more closely investigated experimentally.

#### Step 3: Analysis of the Boolean Model

Once the Boolean model is validated, it can be analyzed with respect to the properties of interest. Again, we give some examples of analysis methods that are particularly useful in application.

First, analysis of the network structure can be conducted utilizing the dependency graph of the Boolean model. Edges in this graph represent component interactions with an observable impact in the model dynamics (see Materials and Methods). Comparison with the dependencies as captured in the ODE model highlights essential regulatory effects while uncovering others not of importance for generating the qualitative dynamical characteristics. A higher level structural analysis focuses on the *feedback circuit functionality* which allows to relate subnetworks to important dynamical phenomena such as multistability and oscillations [11].

With respect to the dynamical behavior, analysis as well as validation can be focused on specific trajectories, but also on global properties [3]. Commonly, an attractor analysis is conducted to obtain an overview of the possible asymptotic behavior. Additionally, a perturbation analysis, both concerning temporary as well as maintained perturbations, often uncovers system characteristics related to robustness and mechanisms governing decision processes.

All mentioned methods have been implemented in various software packages, e.g., GINsim [23] and BoolNet [24]. Sophisticated algorithms for finite transition systems utilizing, e.g., symbolic encodings allow for very efficient analysis. As already discussed, this can provide a much more comprehensive view of the system dynamics than can be achieved in the ODE setting alone. On the one hand, this might yield possibilities to refine the ODE model analysis, e.g., by discovering additional attractors. These might then also be found in the ODE model when exploiting the rough information about their position in state space derived from the discrete representation. On the other hand, comparison and integration of the results obtained from both models gives a much more rounded picture of the design and functionality of specific model mechanisms as well as a clearer understanding on the system level.

### Application to the Model BovCycle

We now apply the above described scheme to the ODE model BovCycle [19] of the bovine estrous cycle. The model describes the growth and decay of the follicles and the corpus luteum, as well as the change of the key reproductive hormones, enzymes, and processes over time. It generates hormone profiles of successive estrous cycles with several follicular waves per cycle. The model consists of 10 ODEs and 38 parameters and is described in more detail in the Materials and Methods section.

#### Step 0: Preliminaries and Assumptions

The model is an autonomous system of ODEs normalized in such a way that all variables take values in [0, 1]. This has been achieved as in [15] by running a simulation of the unscaled ODE system over a time interval *I* and then using the maximum values $y_{i}^{\text{max}}={\text{max}}_{t\in I}y_{i}(t)$ to rescale all variables to $y_{i}/y_{i}^{\text{max}}$.

The right hand sides include only terms representing mass action or Hill kinetics, thus they consist only of sums and products of monotone functions taking values in [0, 1]. Hence, we can proceed with Step 1.

#### Step 1: Translating the ODE System into a Logical Model by an Euler-like Discretization

The discrete functions *h*_*i*_(*x*) take the following form:

$$\begin{array}{cc} h_{\text{GnRH}}\left(x_{\text{P4}},x_{\text{E2}},x_{\text{GnRH}}\right) & =m_{\text{E2},\text{P4}}^{\text{GnRH}}\cdot \left(1-x_{\text{P4}}\right)\cdot x_{\text{E2}}-c_{\text{GnRH}}\cdot x_{\text{GnRH}},\\ h_{\text{FSH}}\left(x_{\text{Inh}},x_{\text{FSH}}\right) & =m_{\text{Inh}}^{\text{FSH}}\cdot \left(1-x_{\text{Inh}}\right)-c_{\text{FSH}}\cdot x_{\text{FSH}},\\ h_{\text{LH}}\left(x_{\text{P4}},x_{\text{GnRH}},x_{\text{LH}}\right) & =m_{\text{GnRH},P4}^{\text{LH}}\cdot \left(1-x_{\text{P4}}\right)\cdot x_{\text{GnRH}}-c_{\text{LH}}\cdot x_{\text{LH}},\\ h_{\text{Foll}}\left(x_{\text{FSH}},x_{\text{P4}},x_{\text{LH}},x_{\text{Foll}}\right) & =m_{\text{FSH}}^{\text{Foll}}\cdot x_{\text{FSH}}(1+m_{\text{Foll}}^{\text{Foll}}\cdot x_{\text{Foll}})-(m_{\text{P4}}^{\text{Foll}}\cdot x_{\text{P4}}+m_{\text{LH}}^{\text{Foll}}\cdot x_{\text{LH}})\cdot x_{\text{Foll}},\\ h_{\text{CL}}\left(x_{\text{LH}},x_{\text{Foll}},x_{\text{IOF}},x_{\text{CL}}\right) & ={\text{SF}}_{\text{CL}}\cdot m_{\text{LH}}^{\text{Foll}}\cdot x_{\text{LH}}\cdot x_{\text{Foll}}+m_{\text{CL}}^{\text{CL}}\cdot x_{\text{CL}}-m_{\text{IOF}}^{\text{CL}}\cdot x_{\text{IOF}}\cdot x_{\text{CL}},\\ h_{\text{P4}}\left(x_{\text{CL}},x_{\text{P4}}\right) & =k_{\text{CL}}^{\text{P4}}\cdot x_{\text{CL}}-c_{\text{P4}}\cdot x_{\text{P4}},\\ h_{\text{E2}}\left(x_{\text{Foll}},x_{\text{E2}}\right) & =k_{\text{Foll}}^{\text{E2}}\cdot x_{\text{Foll}}-c_{\text{E2}}\cdot x_{\text{E2}},\\ h_{\text{Inh}}\left(x_{\text{Foll}},x_{\text{Inh}}\right) & =k_{\text{Foll}}^{\text{Inh}}\cdot x_{\text{Foll}}-c_{\text{Inh}}\cdot x_{\text{Inh}},\\ h_{\text{PGF}}\left(x_{\text{E2}},x_{\text{P4}},x_{\text{PGF}}\right) & =m_{\text{E2}}^{\text{PGF}}\cdot x_{\text{E2}}\cdot x_{\text{P4}}-c_{\text{PGF}}\cdot x_{\text{PGF}},\\ h_{\text{IOF}}\left(x_{\text{PGF}},x_{\text{CL}},x_{\text{IOF}}\right) & =m_{\text{PGF},\text{CL}}^{\text{IOF}}\cdot x_{\text{PGF}}\cdot x_{\text{CL}}-c_{\text{IOF}}\cdot x_{\text{IOF}}. \end{array}$$

To obtain the Boolean update functions for all components, we now need to evaluate the signs of the function *h*_*i*_(*x*) for all Boolean states *x*. Apart from the component values *x*_*j*_ appearing on the right hand side of the function equations for each *h*_*i*_, the value of *h*_*i*_(*x*) depends on the model parameter values, which are listed in the Materials and Methods section. The resulting truth tables for all Boolean functions are given in Tables 1 to 10.

**Table 1:** Updates for *x*_GnRH_ ∈ {0, 1}.

**$x_{\text{P4}}^{k}$**	**$x_{\text{E2}}^{k}$**	**$x_{\text{GnRH}}^{k}$**	***h*_GnRH_**	**sgn(*h*_GnRH_)**	**$x_{\text{GnRH}}^{k+1}$**
0	0	0	0	0	0
0	0	1	−*c*_GnRH_	-1	0
0	1	0	$m_{\text{E2},\text{P4}}^{\text{GnRH}}$	1	1
0	1	1	$m_{\text{E2},\text{P4}}^{\text{GnRH}}-c_{\text{GnRH}}$	1	1
1	0	0	0	0	0
1	0	1	−*c*_GnRH_	-1	0
1	1	0	0	0	0
1	1	1	−*c*_GnRH_	-1	0

**Table 2:** Updates for *x*_FSH_ ∈ {0, 1}.

**$x_{\text{Inh}}^{k}$**	**$x_{\text{FSH}}^{k}$**	***h*_FSH_**	**sgn(*h*_FSH_)**	**$x_{\text{FSH}}^{k+1}$**
0	0	$m_{\text{Inh}}^{\text{FSH}}$	1	1
0	1	$m_{\text{Inh}}^{\text{FSH}}-c_{\text{FSH}}$	1	1
1	0	0	0	0
1	1	−*c*_FSH_	-1	0

**Table 3:** Updates for *x*_LH_ ∈ {0, 1}.

**$x_{\text{P4}}^{k}$**	**$x_{\text{GnRH}}^{k}$**	**$x_{\text{LH}}^{k}$**	***h*_LH_**	**sgn(*h*_LH_)**	**$x_{\text{LH}}^{k+1}$**
0	0	0	0	0	0
0	0	1	−*c*_LH_	-1	0
0	1	0	$m_{\text{GnRH},\text{P4}}^{\text{LH}}$	1	1
0	1	1	$m_{\text{GnRH},\text{P4}}^{\text{LH}}-c_{\text{LH}}$	1	1
1	0	0	0	0	0
1	0	1	−*c*_LH_	-1	0
1	1	0	0	0	0
1	1	1	−*c*_LH_	-1	0

**Table 4:** Updates for *x*_Foll_ ∈ {0, 1}.

**$x_{\text{FSH}}^{k}$**	**$x_{\text{P4}}^{k}$**	**$x_{\text{LH}}^{k}$**	**$x_{\text{Foll}}^{k}$**	***h*_Foll_**	**sgn(*h*_Foll_)**	**$x_{\text{Foll}}^{k+1}$**
0	0	0	0	0	0	0
0	0	0	1	0	0	1
0	0	1	0	0	0	0
0	0	1	1	$-m_{\text{LH}}^{\text{Foll}}$	-1	0
0	1	0	0	0	0	0
0	1	0	1	$-m_{\text{P4}}^{\text{Foll}}$	-1	0
0	1	1	0	0	0	0
0	1	1	1	$-m_{\text{P4}}^{\text{Foll}}-m_{\text{LH}}^{\text{Foll}}$	-1	0
1	0	0	0	$m_{\text{FSH}}^{\text{Foll}}$	1	1
1	0	0	1	$m_{\text{FSH}}^{\text{Foll}}(1+m_{\text{Foll}}^{\text{Foll}})$	1	1
1	0	1	0	$m_{\text{FSH}}^{\text{Foll}}$	1	1
1	0	1	1	$m_{\text{FSH}}^{\text{Foll}}(1+m_{\text{Foll}}^{\text{Foll}})-m_{\text{LH}}^{\text{Foll}}$	-1	0
1	1	0	0	$m_{\text{FSH}}^{\text{Foll}}$	1	1
1	1	0	1	$m_{\text{FSH}}^{\text{Foll}}(1+m_{\text{Foll}}^{\text{Foll}})-m_{\text{P4}}^{\text{Foll}}$	1	1
1	1	1	0	$m_{\text{FSH}}^{\text{Foll}}$	1	1
1	1	1	1	$m_{\text{FSH}}^{\text{Foll}}(1+m_{\text{Foll}}^{\text{Foll}})-m_{\text{P4}}^{\text{Foll}}-m_{\text{LH}}^{\text{Foll}}$	-1	0

**Table 5:** Updates for *x*_CL_ ∈ {0, 1}.

**$x_{\text{LH}}^{k}$**	**$x_{\text{Foll}}^{k}$**	**$x_{\text{IOF}}^{k}$**	**$x_{\text{CL}}^{k}$**	***h*_CL_**	**sgn(*h*_CL_)**	**$x_{\text{CL}}^{k+1}$**
0	0	0	0	0	0	0
0	0	0	1	$m_{\text{CL}}^{\text{CL}}$	1	1
0	0	1	0	0	0	0
0	0	1	1	$-m_{\text{IOF}}^{\text{CL}}$	-1	0
0	1	0	0	0	0	0
0	1	0	1	$m_{\text{CL}}^{\text{CL}}$	1	1
0	1	1	0	0	0	0
0	1	1	1	$m_{\text{CL}}^{\text{CL}}-m_{\text{IOF}}^{\text{CL}}$	-1	0
1	0	0	0	0	0	0
1	0	0	1	$m_{\text{CL}}^{\text{CL}}$	1	1
1	0	1	0	0	0	0
1	0	1	1	$m_{\text{CL}}^{\text{CL}}-m_{\text{IOF}}^{\text{CL}}$	-1	0
1	1	0	0	${\text{SF}}_{\text{CL}}\cdot m_{\text{LH}}^{\text{Foll}}$	1	1
1	1	0	1	${\text{SF}}_{\text{CL}}\cdot m_{\text{LH}}^{\text{Foll}}+m_{\text{CL}}^{\text{CL}}$	1	1
1	1	1	0	${\text{SF}}_{\text{CL}}\cdot m_{\text{LH}}^{\text{Foll}}$	1	1
1	1	1	1	${\text{SF}}_{\text{CL}}\cdot m_{\text{LH}}^{\text{Foll}}+m_{\text{CL}}^{\text{CL}}-m_{\text{IOF}}^{\text{CL}}$	-1	0

**Table 6:** Updates for *x*_P4_ ∈ {0, 1}.

**$x_{\text{CL}}^{k}$**	**$x_{\text{P4}}^{k}$**	***h*_P4_**	**sgn(*h*_P4_)**	**$x_{\text{P4}}^{k+1}$**
0	0	0	0	0
0	1	−*c*_P4_	-1	0
1	0	$k_{\text{CL}}^{\text{P4}}$	1	1
1	1	$k_{\text{CL}}^{\text{P4}}-c_{\text{P4}}$	1	1

**Table 7:** Updates for *x*_E2_ ∈ {0, 1}.

**$x_{\text{Foll}}^{k}$**	**$x_{\text{E2}}^{k}$**	***h*_E2_**	**sgn(*h*_E2_)**	**$x_{\text{E2}}^{k+1}$**
0	0	0	0	0
0	1	−*c*_E2_	-1	0
1	0	$k_{\text{Foll}}^{\text{E2}}$	1	1
1	1	$k_{\text{Foll}}^{\text{E2}}-c_{\text{E2}}$	1	1

**Table 8:** Updates for *x*_Inh_ ∈ {0, 1}.

**$x_{\text{Foll}}^{k}$**	**$x_{\text{Inh}}^{k}$**	***h*_Inh_**	sgn(*h*_E2_)	$x_{\text{Inh}}^{k+1}$
0	0	0	0	0
0	1	−*c*_Inh_	-1	0
1	0	$k_{\text{Foll}}^{\text{Inh}}$	1	1
1	1	$k_{\text{Foll}}^{\text{Inh}}-c_{\text{Inh}}$	1	1

**Table 9:** Updates for *x*_PGF_ ∈ {0, 1}.

**$x_{\text{E2}}^{k}$**	**$x_{\text{P4}}^{k}$**	**$x_{\text{PGF}}^{k}$**	***h*_PGF_**	**sgn(*h*_PGF_)**	**$x_{\text{PGF}}^{k+1}$**
0	0	0	0	0	0
0	0	1	−*c*_PGF_	-1	0
0	1	0	0	0	0
0	1	1	−*c*_PGF_	-1	0
1	0	0	0	0	0
1	0	1	−*c*_PGF_	-1	0
1	1	0	$m_{\text{E2}}^{\text{PGF}}$	1	1
1	1	1	$m_{\text{E2}}^{\text{PGF}}-c_{\text{PGF}}$	-1	1

**Table 10:** Updates for *x*_IOF_ ∈ {0, 1}.

**$x_{\text{PGF}}^{k}$**	**$x_{\text{CL}}^{k}$**	**$x_{\text{IOF}}^{k}$**	***h*_IOF_**	**sgn(*h*_IOF_)**	**$x_{\text{IOF}}^{k+1}$**
0	0	0	0	0	0
0	0	1	−*c*_IOF_	-1	0
0	1	0	0	0	0
0	1	1	−*c*_IOF_	-1	0
1	0	0	0	0	0
1	0	1	−*c*_IOF_	-1	0
1	1	0	$m_{\text{PGF},\text{CL}}^{\text{IOF}}$	1	1
1	1	1	$m_{\text{PGF},\text{CL}}^{\text{IOF}}-c_{\text{IOF}}$	-1	1

#### Step 2: Validation of the Boolean Model

An important feature of the underlying biological system is the oscillation of all components signifying the successive estrous cycles. Simulations of the ODE model reproduce this behavior. Now, to check whether the Boolean model captures the behavior of the numerical model, we decided to validate it using the simulations from the latter. This allows us to illustrate approaches to data discretization and to checking conservation of trajectories similarly applicable to validation with experimental data. In a first step, we derive a discrete time series from the ODE simulation. Secondly, we search the asynchronous state transition graph of the Boolean model for a path matching this time series.

We start by running a forward simulation of the ODE model given in the Materials and Methods section, thus providing an approximation of the continuous solution as a time series of real-valued variables. As mentioned, several discretization schemes are available for converting continuous into Boolean data. Here, a threshold is chosen for each continuous variable *y*_*i*_. Depending on whether the continuous variable is below or above this threshold at a certain point in time, the value 0 or 1 is assigned to the corresponding binary variable *x*_*i*_ for this time point. In the following, the thresholds are called *translation thresholds*.

Hill functions explicitly contain threshold values for some of the continuous variables. In most cases, however, the same continuous variable occurs in several Hill functions, but with different threshold values. Thus, some averaging must be applied. In addition, some variables do not exert their influence via Hill functions but directly, e.g., in terms of mass action kinetics. For these variables, their mean value over time can be used as threshold. Finally, the following rules are applied to derive the translation thresholds.

(i) For each variable, its average value over time is calculated.
(ii) For each variable that exerts an influence via at least one Hill function, the mean of the thresholds in these Hill functions is calculated.
(iii) The mean between the values from (i) and (ii) or, if (ii) does not apply, only the value from (i) is used as translation threshold.

**Fig 1:**
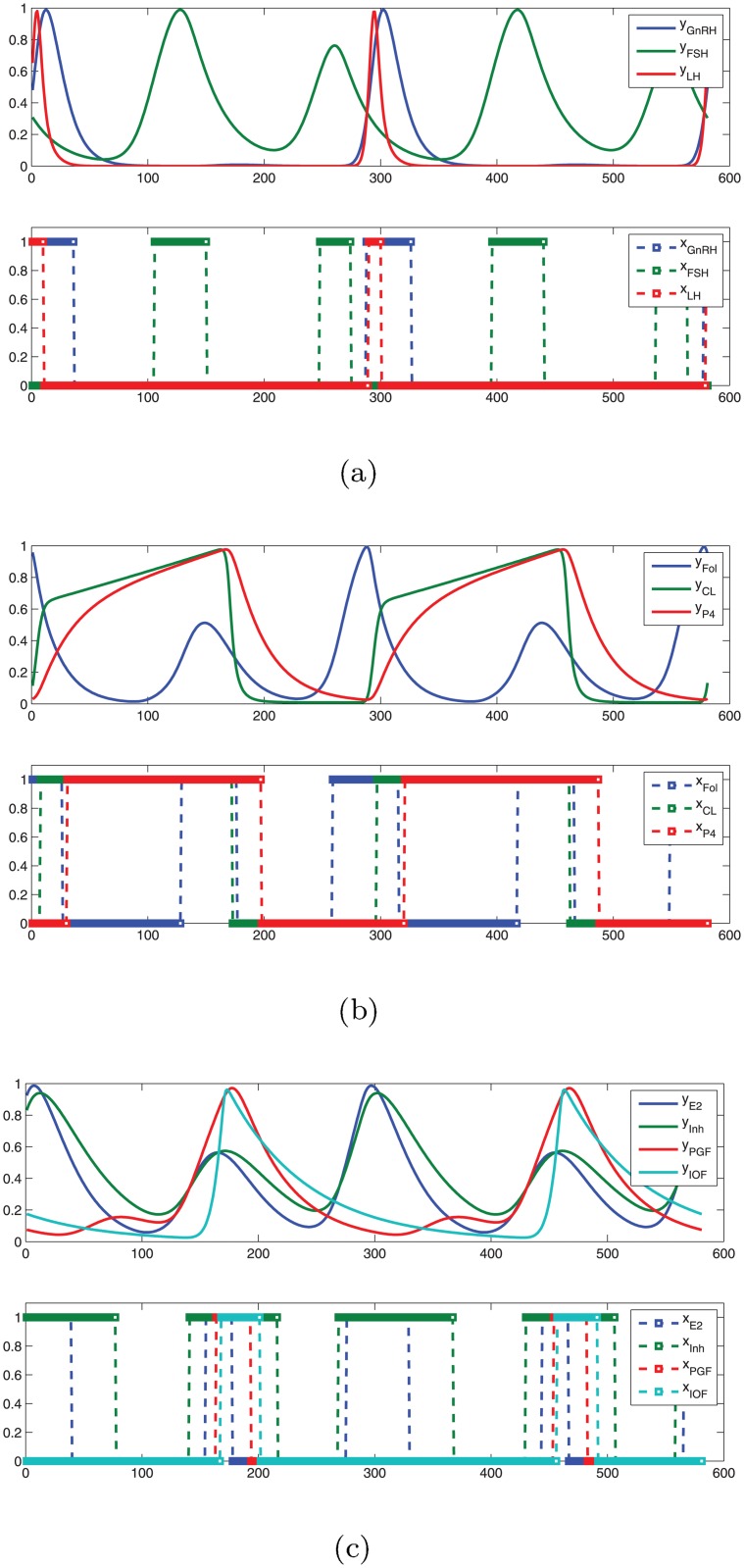
ODE simulation from the model BovCycle and the generated discrete time series. This time series is used to create a sequence of binary states for the validation of the discrete model. (a) Simulation output for GnRH, FSH, and LH. (b) Simulation output for Fol, CL, and P4. (c) Simulation output for E2, Inh, PGF, and IOF.

Averaging in (iii) is reasonable because a Hill-threshold does not need to be fully reached by a continuous variable in order to have a regulatory impact on the other variables. Based on these translation thresholds, the binary time series is generated, and repeating entries are removed. The derived sequence of states is depicted in Fig 1, and explicitly given in Table 11. Double-checking the discretization result, we also find that it matches the observed qualitative behavior of the underlying biological system.

**Table 11:** Discrete sequence of states generated from the time series of the continuous model.

***x*_GnRH_**	***x*_FSH_**	***x*_LH_**	***x*_Foll_**	***x*_CL_**	***x*_P4_**	***x*_E2_**	***x*_Inh_**	***x*_PGF_**	***x*_IOF_**	**Event**
1	0	1	1	0	0	1	1	0	0	*x*_CL_ ↑
1	0	1	1	1	0	1	1	0	0	*x*_LH_ ↓
1	0	0	1	1	0	1	1	0	0	*x*_Foll_ ↓
1	0	0	0	1	0	1	1	0	0	*x*_P4_ ↑
1	0	0	0	1	1	1	1	0	0	*x*_GnRH_ ↓
0	0	0	0	1	1	1	1	0	0	*x*_E2_ ↓
0	0	0	0	1	1	0	1	0	0	*x*_Inh_ ↓
0	0	0	0	1	1	0	0	0	0	*x*_FSH_ ↑
0	1	0	0	1	1	0	0	0	0	*x*_Foll_ ↑
0	1	0	1	1	1	0	0	0	0	*x*_Inh_ ↑
0	1	0	1	1	1	0	1	0	0	*x*_FSH_ ↓
0	0	0	1	1	1	0	1	0	0	*x*_E2_ ↑
0	0	0	1	1	1	1	1	0	0	*x*_PGF_ ↑
0	0	0	1	1	1	1	1	1	0	*x*_IOF_ ↑
0	0	0	1	1	1	1	1	1	1	*x*_CL_ ↓
0	0	0	1	0	1	1	1	1	1	*x*_Foll_ ↓
0	0	0	0	0	1	1	1	1	1	*x*_E2_ ↓
0	0	0	0	0	1	0	1	1	1	*x*_PGF_ ↓
0	0	0	0	0	1	0	1	0	1	*x*_P4_ ↓
0	0	0	0	0	0	0	1	0	1	*x*_IOF_ ↓
0	0	0	0	0	0	0	1	0	0	*x*_Inh_ ↓
0	0	0	0	0	0	0	0	0	0	*x*_FSH_ ↑
0	1	0	0	0	0	0	0	0	0	*x*_Foll_ ↑
0	1	0	1	0	0	0	0	0	0	*x*_Inh_ ↑
0	1	0	1	0	0	0	1	0	0	*x*_FSH_ ↓
0	0	0	1	0	0	0	1	0	0	*x*_E2_ ↑
0	0	0	1	0	0	1	1	0	0	*x*_GnRH_ ↑
1	0	0	1	0	0	1	1	0	0	*x*_LH_ ↑
1	0	1	1	0	0	1	1	0	0	

To check whether this state sequence is generated by the Boolean model, we search for a path in the state transition graph that visits the given states in the correct order. Hereby, we allow for intermediate states since not every system state might be captured by the time series. This can be done, e.g., by using a path search in GINsim [23] or via model checking as in [22]. We find indeed that such a path exists, i.e., the Boolean model is capable of generating the desired behavior.

#### Step 3: Analysis of the Boolean Model

**Fig 2:**
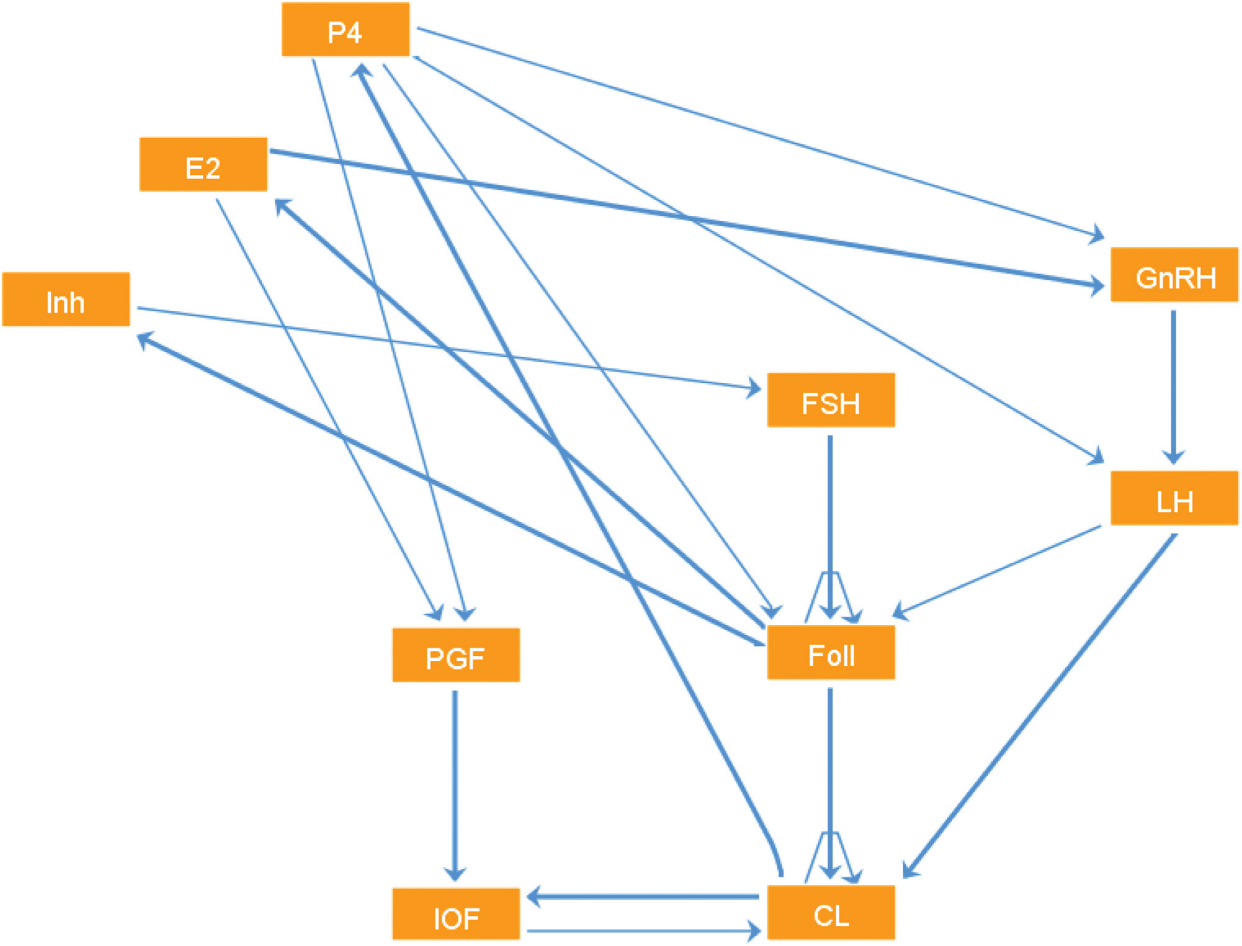
Dependency graph of the Boolean model for the bovine estrous cycle. This graph directly follows from the discrete updates calculated in Step 1, which are derived from the right hand sides of the ODE model. Implemented in GINsim [23].

We start with a structural analysis of the Boolean model, having a closer look at the dependency graph (Fig 2) which is directly derived from the listed truth tables of the Boolean functions. Looking at the equations for the functions *h*_*i*_ derived in Step 1, we see the potential for self-regulation for each component in the decay terms incorporated in the differential equations.

However, a closer look at the truth tables shows that these decay terms in almost all cases need not be explicitly modeled in the Boolean setting. As an example consider the regulation of FSH, where in the left two columns of Table 2 the two variables Inh and FSH as given in the ODE are listed. However, looking at the rightmost column and comparing the first two with the last two rows, we can easily see that the value of the FSH update function only depends on the value of Inh. The decay term included in the FSH ODE thus has no functional impact on this level of abstraction.

There are two exceptions to this observation, the components Foll and CL. Further analysis shows that both exhibit an observable self-activating effect, which again can already be seen in the respective differential equations. Interestingly, both also display a self-inhibition, e.g., in the state where all their other regulators are active, as can be seen in Tables 4 and 5. This indicates that certain mechanisms driving the system function potentially rely on these self-inhibitory effects that might be implemented either as an active inhibition or via a simple decay.

Next, we have a closer look at the shortest path in the state transition graph traversing the time series given in Table 11. Most of the given states can be reached by a direct transition from the preceding state, meaning there is an edge between these two states in the state transition graph. Exceptions are the second and the third transition in Table 11. Although the states also only differ in one component value, it takes several intermediate steps to get from one state to the next, i.e., there are only paths of length greater than 1 linking the states in the state transition graph. In the second transition, e.g., we want to see a down-regulation of *x*_LH_. Comparing the current state with the truth table of the *x*_LH_-update function, we see that to obtain this effect first down-regulation of *x*_GnRH_ or up-regulation of *x*_P4_ needs to occur, which in turn is driven by other regulators. Activity level changes in components other than *x*_LH_ need then to be reversed after adapting the activity of *x*_LH_ to match the given measurement. Thus, we uncovered a hidden oscillation of a subnetwork driving the system along the observed trajectory. Similar results can be obtained for the down-regulation of *x*_Foll_ in the third transition.

Lastly, we want to address a phenomenon observed in the continuous setting. A remarkable property of the ODE model for the bovine estrous cycle is the stability of the limit cycle solution with respect to perturbations of state variables and parameter values. For the continuous case, local stability has been proven [19], but the proof cannot be extended to show global stability. For the discrete model, however, we can perform a more global analysis.

To this end, we conducted an attractor analysis considering the state transition graph of the model in its entirety using the software GINsim [23]. As a first result, we found that the network does not have any steady states. This already indicates strongly that the system is tailored to generate stable oscillatory behavior.

Further analysis shows that the system has a single attractor of high complexity, which contains the time series given in Table 11 encoding the normal estrous cycle. Since the entire state space constitutes the basin of attraction for this attractor, the cyclic path encoding the time series is reachable from every state of the system. This again indicates a high degree of stability, since the system can recover its standard behavior after an arbitrary state perturbation.

The analysis steps shown here already yield several interesting results that could be investigated much further. A circuit functionality analysis [25] could shed light on the question of why there is only a single attractor and provide a better understanding of what modules of the network may be responsible for certain characteristics of the stable oscillation. In other applications, one could also investigate the influence of e.g. knock-outs/ins on the model behavior to identify key components. A comparative analysis of such scenarios of the logical and ODE model might also further clarify the relation between the two models.

## Materials and Methods

In this section, we introduce the concepts and notations of ordinary differential equation models and logical models. In addition, we briefly introduce the software GINsim that is used for validation of the logical model. Moreover, we present the equations and parameter values for the model BovCycle, which is used as illustrative example in the paper.

### ODE Modeling

An ordinary differential equation (ODE) model is based on a state-vector $y(t)=(y_{1}(t),\ldots ,y_{n}(t))\in {\mathbb{R}}^{n}$ of time-dependent, continuous variables representing the concentrations or activation levels of certain system components, e.g., chemical compounds, populations of cells or animals. Their change over time is described by the first derivatives, *y*′(*t*) = *f*(*t*, *y*, *p*), modeled by a parameter-dependent function *f*(*t*, *y*, *p*) = (*f*_1_(*t*, *y*, *p*), …, *f*_*n*_(*t*, *y*, *p*)). Here $p\in {\mathbb{R}}^{q}$ denotes the vector of (unknown) model parameters values.

ODEs have become a standard tool in systems biology to model complex systems enriched in non-linear motifs like feedback and feed-forward loops [26]. Construction and analysis of ODE models for such systems, however, face a number of difficulties. Two fundamental questions have to be addressed: the derivation of model equations and the estimation of model parameters. For the construction of model equations, canonical modeling frameworks like mass-action kinetic, Hill kinetics or power-law modeling are often used.

Parameter estimation, however, is a computationally intensive task that requires large amounts of quantitative experimental data (dose-response-experiments, time series data, etc.) and sophisticated optimization algorithms. Alternative methods for exploring the dynamical behavior of ODE models are therefore desirable.

### Logical Modeling

A logical network model is based on a set of discrete variables {*x*_1_, …, *x*_*n*_},n∈ℕ, representing the network components {1, …, *n*}. Each variable *x*_*i*_ can take values in a set {0, 1, …, *m*_*i*_},$m_{i}\in \mathbb{N}$, of integers interpreted as the different activity levels of the component. In the simplest case, all variables only take values 0 and 1, resulting in a Boolean network. The state space of the network is given by the set *S* : = {0, …, *m*_1_} × … × {0, …, *m*_*n*_}. All interactions between the different components generating the system behavior are then modeled by a logical function *g* = (*g*_1_, …, *g*_*n*_) : *S* → *S*, where each coordinate function *g*_*i*_ captures the way component *x*_*i*_ is regulated in a given system state. The dependencies of the coordinate functions on particular components are captured in a dependency or interaction graph where the vertices represent the network components, and there is an edge from *i* to *j* if *f*_*j*_ depend on *x*_*i*_. Edges can also carry a sign indicating whether the regulatory effect is positive or negative (see e.g. [3]). It indicates the existence of at least one network state, where the change in the regulator’s activity level generates a similar, in the case of a positive interaction, or opposite, in the case of a negative interaction, effect on the target component.

The dynamics of the network can be represented by a finite directed state transition graph with vertex set *S* and edges representing state transitions derived from *g* according to a certain update strategy. The simplest strategy, called synchronous update, is to define the successor of a state *s* as *g*(*s*). A strategy taking into account the different time delays associated to different regulatory events is the so-called asynchronous update where only one component value is changed per update step: In the Boolean case, we get that $\hat{s}$ is a successor of *s* if there exists *i* ∈ {1, …, *n*} such that $\hat{s}=g_{i}(s)\neq s_{i}$ and ${\hat{s}}_{j}=s_{j}$ for all *j* ≠ *i* [27]. This results in a non-deterministic state transition graph usually comprising more realistic trajectories then generated by the synchronous update.

### Discrete Analysis Tools

All validation and analysis steps presented for the Boolean model of the bovine estrous cycle can be conducted using GINsim [23], a software for simulation and analysis of qualitative models. It is based on the multilevel logical formalism introduced in [27], which includes the Boolean formalism used in this paper. In GINsim, among other functionalities, the state transition graph of a model can be calculated and then analyzed using a number of tools. Included are efficient algorithms for the computation of steady states, shortest path search in the state transition graph and computation of the strongly connected component graph allowing to identify cyclic attractors and basins of attraction.

### The ODE Model BovCycle

As in humans, the bovine endocrine system consists of several glands and regulates the periodic changes of multiple substances necessary for reproduction. In every cycle, hormones are secreted from the hypothalamic-pituitary-gonadal axis into the bloodstream, where they distribute and influence several functions in the body. Their most important task in reproduction is to regulate processes in the ovaries, where follicles and corpus luteum develop. These produce steroids that are released into the blood and from therein regulate the processes in the hypothalamic-pituitary-gonadal axis. The complex interplay of these multiple components generates the cyclic hormonal changes without external stimuli. The large feedback loop of regulations results in the hormonal cycle.

In the ODE model BovCycle, interactions between biological substances are mostly of regulatory nature and are modeled by Hill functions. An increasing Hill function stands for activation or stimulation, a decreasing Hill function for inhibition. Since Hill functions have a sigmoidal shape, their approximation by discrete variables in a logical model is a reasonable approach. The current model [19], which serves as an example for the discretization method in this paper, contains 10 ODEs and 38 parameters. Model equations and the here used parameter values are listed in Tables 12 and 13. The model represents a reduced version of a more complex model published in [18].

**Table 12:** The model BovCycle consisting of 10 ODEs and 38 parameters.

$\frac{d}{dt}y_{\text{GnRH}}(t)$	=	$m_{\text{E2},\text{P4}}^{\text{GnRH}}\cdot H_{\text{P4},\text{GnRH}}^{-}\left(y_{\text{P4}}(t)\right)\cdot H_{\text{E2},\text{GnRH}}^{+}\left(y_{\text{E2}}(t)\right)-c_{\text{GnRH}}\cdot y_{\text{GnRH}}(t)$
$\frac{d}{dt}y_{\text{FSH}}(t)$	=	$m_{\text{Inh}}^{\text{FSH}}\cdot H_{\text{Inh},\text{FSH}}^{-}(y_{\text{Inh}}(t))-c_{\text{FSH}}\cdot y_{\text{FSH}}(t)$
$\frac{d}{dt}y_{\text{LH}}(t)$	=	$m_{\text{GnRH},P4}^{\text{LH}}\cdot H_{\text{P4},\text{LH}}^{-}(y_{\text{P4}}(t))\cdot H_{\text{GnRH},\text{LH}}^{+}\left(y_{\text{GnRH}}(t)\right)-c_{\text{LH}}\cdot y_{\text{LH}}(t)$
$\frac{d}{dt}y_{\text{Foll}}(t)$	=	$\begin{array}{l} m_{\text{FSH}}^{\text{Foll}}\cdot H_{\text{FSH},\text{Foll}}^{+}\left(y_{\text{FSH}}(t)\right)\cdot \left(1+m_{\text{Foll}}^{\text{Foll}}\cdot H_{\text{Foll},\text{Foll}}^{+}\left(y_{\text{Foll}}(t)\right)\right)\\ \;-\left(m_{\text{P4}}^{\text{Foll}}\cdot H_{\text{P4},\text{Foll}}^{+}(y_{\text{P4}}(t))+m_{\text{LH}}^{\text{Foll}}\cdot H_{\text{LH},\text{Ovul}}^{+}(y_{\text{LH}}(t))\right)\cdot y_{\text{Foll}}(t) \end{array}$
$\frac{d}{dt}y_{\text{CL}}(t)$	=	$\begin{array}{l} {\text{SF}}_{\text{CL}}\cdot m_{\text{LH}}^{\text{Foll}}\cdot H_{\text{LH},\text{Ovul}}^{+}\left(y_{\text{LH}}(t)\right)\cdot y_{\text{Foll}}(t)+m_{\text{CL}}^{\text{CL}}\cdot H_{\text{CL},\text{CL}}^{+}\left(y_{\text{CL}}(t)\right)\\ \;-m_{\text{IOF}}^{\text{CL}}\cdot H_{\text{IOF},\text{CL}}^{+}\left(y_{\text{IOF}}(t)\right)\cdot y_{\text{CL}}(t) \end{array}$
$\frac{d}{dt}y_{\text{P4}}(t)$	=	$k_{\text{CL}}^{\text{P4}}\cdot y_{\text{CL}}(t)-c_{\text{P4}}\cdot y_{\text{P4}}(t)$
$\frac{d}{dt}y_{\text{E2}}(t)$	=	$k_{\text{Foll}}^{\text{E2}}\cdot y_{\text{Foll}}(t)-c_{\text{E2}}\cdot y_{\text{E2}}(t)$
$\frac{d}{dt}y_{\text{Inh}}(t)$	=	$k_{\text{Foll}}^{\text{Inh}}\cdot y_{\text{Foll}}(t)-c_{\text{Inh}}\cdot y_{\text{Inh}}(t)$
$\frac{d}{dt}y_{\text{PGF}}(t)$	=	$m_{\text{E2}}^{\text{PGF}}\cdot H_{\text{E2},\text{PGF}}^{+}(y_{\text{E2}}(t))\cdot H_{\text{P4},\text{PGF}}^{+}(y_{\text{P4}}(t))-c_{\text{PGF}}\cdot y_{\text{PGF}}(t)$
$\frac{d}{dt}y_{\text{IOF}}(t)$	=	$m_{\text{PGF},\text{CL}}^{\text{IOF}}\cdot H_{\text{PGF},\text{IOF}}^{+}(y_{\text{PGF}}(t))\cdot H_{\text{CL},\text{IOF}}^{+}(y_{\text{CL}}(t))-c_{\text{IOF}}\cdot y_{\text{IOF}}(t)$

The notation for the Hill functions $H_{S_{1},S_{2}}^{+/-}(y_{S_{1}}(t))$ is an abbreviation for $H^{+/-}(y_{S_{1}}(t),T_{S_{1}}^{S_{2}},r_{S_{1}}^{S2})$, whereby $H^{-}(y_{S_{1}}(t),T_{S_{1}}^{S_{2}},r_{S_{1}}^{S2})=1-H^{+}(y_{S_{1}}(t),T_{S_{1}}^{S_{2}},r_{S_{1}}^{S2})$.

**Table 13:** BovCycle model parameters and their values which lead to the simulation of an estrous cycle with two follicular waves per cycle.

**No.**	**Symbol**	**Value**	**Unit**
1	$m_{\text{E2},\text{P4}}^{\text{GnRH}}$	5.707	[GnRH]/[t]
2	$T_{\text{P4}}^{\text{GnRH}}$	0.271	[P4]
3	$T_{\text{E2}}^{\text{GnRH}}$	1.127	[E2]
4	*c*_GnRH_	1.223	1/[t]
5	$m_{\text{Inh}}^{\text{FSH}}$	1.044	[FSH]/[t]
6	$T_{\text{Inh}}^{\text{FSH}}$	0.217	[Inh]
7	*c*_FSH_	0.559	1/[t]
8	$m_{\text{GnRH},P4}^{\text{LH}}$	46.647	[LH]/[t]
9	$T_{\text{P4}}^{\text{LH}}$	0.0542	[P4]
10	$T_{\text{GnRH}}^{\text{LH}}$	0.896	[GnRH]
11	*c*_LH_	9.006	1/[t]
12	$m_{\text{FSH}}^{\text{Foll}}$	0.269	[Foll]/[t]
13	$T_{\text{FSH}}^{\text{Foll}}$	0.787	[FSH]
14	$m_{\text{Foll}}^{\text{Foll}}$	3.927	-
15	$T_{\text{Foll}}^{\text{Foll}}$	0.289	[Foll]
16	$m_{\text{P4}}^{\text{Foll}}$	0.79	1/[t]
17	$T_{\text{P4}}^{\text{Foll}}$	0.125	[P4]
18	$m_{\text{LH}}^{\text{Foll}}$	1.7	[1/[t]
19	$T_{\text{LH}}^{\text{Foll}}$	0.881	[LH]
20	SF_CL_	1.2	[CL]
21	$m_{\text{CL}}^{\text{CL}}$	0.0372	[CL]/[t]
22	$T_{\text{CL}}^{\text{CL}}$	0.314	[CL]
23	$m_{\text{IOF}}^{\text{CL}}$	7.534	1/[t]
24	$T_{\text{IOF}}^{\text{CL}}$	1.035	[IOF]
25	$k_{\text{CL}}^{\text{P4}}$	0.564	[P4]/[t]
26	*c*_P4_	0.533	1/[t]
27	$k_{\text{Foll}}^{\text{E2}}$	1.009	[E2]/[t]
28	*c*_E2_	0.72	1/[t]
29	$k_{\text{Foll}}^{\text{Inh}}$	0.644	[Inh]/[t]
30	*c*_Inh_	0.368	1/[t]
31	$m_{\text{E2}}^{\text{PGF}}$	1.291	[PGF]/[t]
32	$T_{\text{E2}}^{\text{PGF}}$	0.221	[E2]
33	$T_{\text{P4}}^{\text{PGF}}$	0.969	[P4]
34	*c*_PGF_	0.356	1/[t]
35	$m_{\text{PGF},\text{CL}}^{\text{IOF}}$	12.269	[IOF]/[t]
36	$T_{\text{PGF}}^{\text{IOF}}$	1.282	[PGF]
37	$T_{\text{CL}}^{\text{IOF}}$	0.639	[CL]
38	*c*_IOF_	0.215	1/[t]

Hill exponents are fixed as $r_{\text{E2}}^{\text{GnRH}}=r_{\text{Inh}}^{\text{FSH}}=r_{\text{P4}}^{\text{Foll}}=r_{\text{IOF}}^{\text{CL}}=r_{\text{P4}}^{\text{PGF}}=r_{\text{PGF}}^{\text{IOF}}=r_{\text{CL}}^{\text{IOF}}=5$, all other Hill exponents are set to 2.

## Discussion

In this paper, we presented a generic method for translating an ODE into a Boolean model based on an Euler approximation of the continuous behavior. This straightforward approach is augmented by a subsequent mandatory validation step ensuring that the Boolean network is suitable for describing the properties of interest, either with respect to the ODE model or the underlying biological system. Analysis then allows to address questions not easily accessible in the context of an elaborate ODE model and provides an alternative view on system characteristics, as we illustrated in our case study.

The Euler step is executed and evaluated to derive the qualitative update functions from the ODEs. Mathematically, it captures the behavioral tendencies of the continuous systems in a given state via the derivative signs. Normalization and monotonicity constraints of the continuous regulatory functions ensure that, regardless of the step size, these tendencies capture a long-term effect rather than some initial fluctuations in the transient phase. To account for the fact that, in a continuous system of interacting components, the asymptotic component behavior does not always play out along a trajectory due to regulatory influences, we chose the asynchronous update for the resulting Boolean models. Here, a component update is not necessarily carried out on a specific trajectory if other component updates compete. Based on these observations, the derived Boolean model can be expected to capture the qualitative aspects of its ODE counterpart. However, property conservation is not guaranteed. The validation step of our procedure is necessary to double-check the behavioral match for the dynamical phenomenon of interest. Depending on the modeling objective, the validation strategies presented here could be complemented by other methods, e.g. an explicit analysis on conservation of transition sequences in both the ODE and the Boolean model.

In the Supporting Information (S1 File), we present an alternative method to transform a system of ODEs fulfilling the preliminaries of Step 0 into a Boolean network. This method is inspired by a translation scheme presented in [15] where a specific coarse-grained limit of an ODE system leads to the formulation of a Boolean network. In [15], the results of a bifurcation analysis are used to derive a sequence of stationary states. In the stationary states, variables can be directly described by the Goldbeter-Koshland function, a function with two limiting states. Its approximation by Heaviside functions allows to derive an interaction matrix. Together with a state vector of thresholds, this determines the transition rules between the states and leads to a system whose trajectory is a sequence of Boolean states.

Our method described in the Supporting Information (S1 File) first approximates the unregulated variables or functions in the right hand-sides as integers. Instead of considering bifurcation points, our alternative method takes into account all possible combinations of integers. Due to the approximation, for all cases, the variables can be directly described by linear ODEs which are solved analytically. Then, to overcome the time dependency, the time limit of the system is considered in order to derive the update functions. We show that, for any system of ODEs fulfilling the preliminaries, the therein proposed method leads to the same Boolean network as the described Euler-like transformation.

From the significant abstraction step it is clear that the resulting Boolean network cannot capture all intricacies of the ODE model, and even much less of the underlying biological system. The lack of rigorous proofs for property conservation across formalisms might be considered as main limitation of our proposed method. However, such proofs would certainly require much more severe restrictions on the class of ODE models to be transformed. Here, our aim was to show the easy applicability of the proposed procedure to a broad class of models.

Due to the above considerations, the validation step is of major importance to verify that key aspects of the system are reproduced and should be handled with care. In particular, preprocessing of the data, e.g. discretization of quantitative measurements, can influence the results [20, 21]. The choice of a suitable preprocessing technique and subsequent evaluation, e.g. a sensitivity analysis, can contribute to ensure rigorous results. Generally, a closer look at the reason for a possibly failed validation step can lead to uncover crucial mechanisms not yet captured in the model. Comparison of the validated ODE model with the derived logical model may also help to uncover quantitative properties of functional importance for the biological regulation mechanisms.

In future research, our proposed approach can be adapted and extended to achieve a better fit. A first easy alteration could concern the evaluation of the regulatory effects within the Euler step. Here, one can introduce a threshold for positive and negative tendencies to become effective in the qualitative setting rather than to factor in each non-zero effect.

Furthermore, a higher resolution of activity levels might be very useful, leading to multi-valued instead of Boolean models. For this, the Euler step could be adapted in a way that the update function, instead of directly calculating the next value for a component, only provides the augmentation of the component, which is set to -1 in case of negative derivative of the right hand side, and zero in case that the derivative is zero. Additionally, one could define the update function such that it depends not only on the sign but also, with the help of thresholds, on the value of the derivative. Provided that a reasonable validation is applied, the multi-valued formalism might then allow functionally necessary complexity while keeping the available advantages for analysis.

## Supporting Information

S1 File An Alternative Discretization Method. (PDF)
