# Supplementary material for: Complementing ODE-Based System Analysis Using Boolean Networks Derived from an Euler-Like Transformation
Source: PLoS One. 2015 Oct 23;10(10):e0140954. doi: 10.1371/journal.pone.0140954 (PMC4619740; doi:10.1371/journal.pone.0140954)
Supplement: S1 File — (PDF) [file pone.0140954.s001.pdf]

Complementing ODE-based System Analysis Using Boolean Networks Derived from an Euler-like Transformation

Claudia Stötzel[1], Susanna Röblitz[1,2,¤a,*], Heike Siebert[2]

1 Mathematics for Life and Materials Sciences, Zuse Institute Berlin, Berlin, Germany
2 Dep. of Mathematics and Computer Science, Freie Universität Berlin, Berlin, Germany

¤a Takustr. 7, 14195 Berlin, Germany
*** susanna.roeblitz@zib.de**

Supporting Information

S1 An Alternative Discretization Method

In our paper, we presented a method for translating an ODE model into a discrete model. The update functions in the discrete model were derived from an Euler-like step. In this section, we describe an alternative discretization method that leads to the same update functions but allows for a different interpretation, as addressed in the discussion of the paper.

Discretization by Limit Considerations. Similar to the method described in the paper, continuous variables $y_1, \ldots, y_n$ in the right hand side function $f(y)$ are replaced by their discrete counterparts $x_1, \ldots, x_n$ to map the right hand sides $f_i(y)$ to their discrete counterparts $\hat{h}_i(x)$. This time, however, an exception is made for variables y_i in the right hand side functions $f_i(y)$: monotonically increasing functions $F_{i,i}(y_i)$ are replaced by y_i, whereas monotonically decreasing functions $F_{i,i}(y_i)$ are replaced by $1 - y_i$. The mapping thus takes the form

$$f_i(F_{i,1}(y_1), \ldots, F_{i,n}(y_n)) \mapsto f_i(\underbrace{TF_{i,1}(x_1)}_{x_1 \text{ or } 1-x_1}, \ldots, \underbrace{TF_{i,i}(y_i)}_{y_i \text{ or } 1-y_i}, \ldots, \underbrace{TF_{i,n}(x_n)}_{x_n \text{ or } 1-x_n}) =: \hat{h}_i(x_1, \ldots, y_i, \ldots, x_n)$$

for $i = 1, \ldots, n$. The right hand sides $f_i : \mathbb{R}^n \to \mathbb{R}$ of the ODE system are thus mapped to functions $\hat{h}_i : \{0,1\}^{n-1} \times \mathbb{R} \to \mathbb{R}$, $i = 1, \ldots, n$. This leads to n linear ODEs in the form

$$y_i' = \hat{h}_i(x_1, \ldots, x_{i-1}, y_i, x_{i+1}, \ldots, x_n) = c_{i1} - c_{i2} \cdot y_i, \quad y_i(t_0) = y_i^0, \tag{1}$$

with constant terms c_{i1} and c_{i2}. These constants depend on the values of the discrete variables $x_1, \ldots, x_{i-1}, x_{i+1} \ldots, x_n$ and the model parameters p.

These linear ODEs are now solved analytically and, for all combinations of values c_{i1} and c_{i2}, the limit as $t \to \infty$ is considered to derive the update rule. The initial value y_i^0 is identified with the current state of the discrete system, x_i^k. In particular, for $c_{i2} \neq 0$, the analytic solution for such a linear ODE is given by

$$y_i(t) = \frac{c_{i1}}{c_{i2}} + \left(y_i(t_0) - \frac{c_{i1}}{c_{i2}} \right) \exp(-c_{i2}(t - t_0)).$$

For $c_{i2} = 0$, we have $y_i' = c_{i1}$, and thus the analytic solution

$$y_i(t) = c_{i1}(t - t_0) + y_i(t_0).$$

Now, to get rid of the time-dependence, we consider the limit of $y_i(t)$ as t tends to infinity. Depending on the values of c_{i1}, c_{i2} (smaller than, equal to, or larger than zero), and $y_i(t_0)$ (0 or 1), 18 different cases must be considered, compare S1 Table A. The update rule for the Boolean variable x_i^k in step $k+1$ is then chosen according to

$$x_i^{k+1} = \begin{cases} 1 & \text{, if } \lim_{t\to\infty} y_i(t) > x_i^k, \\ x_i^k & \text{, if } \lim_{t\to\infty} y_i(t) = y_i(t_0) = x_i^k, \\ 0 & \text{, if } \lim_{t\to\infty} y_i(t) < x_i^k. \end{cases} \tag{2}$$

Comparison with the Euler Method Note that in the Euler method, the right hand sides $f_i : \mathbb{R}^n \to \mathbb{R}$ of the ODE system are mapped to functions $h_i : \{0,1\}^n \to \mathbb{R}$. For comparison with the alternative method, these functions can be written as

$$h_i(x_1, \ldots, x_n) = c_{i1} - c_{i2} \cdot x_i,$$

where the constant terms c_{i1} and c_{i2} are the same as above.

In S1 Table A, the transformation from an ODE to a discrete model is performed with both methods. One can observe that, in any case, both methods lead to the same update. Thus, for the ODE models that fulfill the assumptions described in Step 0 of our paper, the two discretization methods lead to the same binary model.

Table A. Comparison of the results of the two discretization methods for the update rule of a discrete variable x^k.

Cases			Limit method		Euler method		
c_1	c_2	$y(t_0)$ or x^k	$y(t) = \begin{cases} \frac{c_1}{c_2} + \left(y(t_0) - \frac{c_1}{c_2}\right) e^{-c_2(t-t_0)} \\ c_1(t-t_0) + y(t_0), \text{ if } c_2 = 0 \end{cases}$	$x_{\hat{h}}^{k+1}$	$h(x^k)$	$sgn(h(x^k))$	x_h^{k+1}
0	0	0	$y(t) = y(t_0) \begin{cases} = 0 \\ = 1 \end{cases}$	0	0	0	0
		1		1	0	0	1
0	> 0	0	$y(t) = y(t_0)e^{-c_2(t-t_0)} \xrightarrow{t\to\infty} 0$	0	0	0	0
		1		0	$-c_2$	< 0	0
0	< 0	0	$y(t) = y(t_0)e^{-c_2(t-t_0)} \begin{cases} = 0 \\ \xrightarrow{t\to\infty} \infty \end{cases}$	0	0	0	0
		1		1	$-c_2$	> 0	1
> 0	0	0	$y(t) = c_1(t-t_0) + y(t_0) \xrightarrow{t\to\infty} \infty$	1	c_1	> 0	1
		1		1	c_1	> 0	1
> 0	> 0	0	$\frac{c_1}{c_2} + (y(t_0) - \frac{c_1}{c_2})\underbrace{e^{-c_2(t-t_0)}}_{\xrightarrow{t\to\infty} 0} \xrightarrow{t\to\infty} \frac{c_1}{c_2} \begin{cases} > 0 \\ \begin{cases} = 1, & c_1 = c_2 \\ > 1, & c_1 > c_2 \\ < 1, & c_1 < c_2 \end{cases} \end{cases}$	1	c_1	> 0	1
		1		$\begin{cases} 1 \\ 1 \\ 0 \end{cases}$	$c_1 - c_2$	$\begin{cases} = 0, & c_1 = c_2 \\ > 0, & c_1 > c_2 \\ < 0, & c_1 < c_2 \end{cases}$	$\begin{cases} 1 \\ 1 \\ 0 \end{cases}$
> 0	< 0	0	$y(t) = \frac{c_1}{c_2} + \underbrace{\left(y(t_0) - \frac{c_1}{c_2}\right)}_{>0} \underbrace{e^{-c_2(t-t_0)}}_{\xrightarrow{t\to\infty} \infty} \xrightarrow{t\to\infty} \infty$	1	c_1	> 0	1
		1		1	$c_1 - c_2$	> 0	1
< 0	0	0	$y(t) = c_1(t-t_0) + y(t_0) \xrightarrow{t\to\infty} -\infty$	0	c_1	< 0	0
		1		0	c_1	< 0	0
< 0	> 0	0	$y(t) = \frac{c_1}{c_2} + (y(t_0) - \frac{c_1}{c_2})\underbrace{e^{-c_2(t-t_0)}}_{\xrightarrow{t\to\infty} 0} \xrightarrow{t\to\infty} -\left\lvert\frac{c_1}{c_2}\right\rvert$	0	c_1	< 0	0
		1		0	$c_1 - c_2$	< 0	0
< 0	< 0	0	$\frac{c_1}{c_2} + \underbrace{\left(y(t_0) - \frac{c_1}{c_2}\right)}_{0, \text{ or } >0, \text{ or } <0} \underbrace{e^{-c_2(t-t_0)}}_{\xrightarrow{t\to\infty} \infty} \begin{cases} \xrightarrow{t\to\infty} -\infty \\ \begin{cases} \xrightarrow{t\to\infty} \frac{c_1}{c_2} = 1, & \lvert c_1\rvert = \lvert c_2\rvert \\ \xrightarrow{t\to\infty} -\infty, & \lvert c_1\rvert > \lvert c_2\rvert \\ \xrightarrow{t\to\infty} \infty, & \lvert c_1\rvert < \lvert c_2\rvert \end{cases} \end{cases}$	0	c_1	< 0	0
		1		$\begin{cases} 1 \\ 0 \\ 1 \end{cases}$	$c_1 - c_2$	$\begin{cases} = 0, & \lvert c_1\rvert = \lvert c_2\rvert \\ < 0, & \lvert c_1\rvert > \lvert c_2\rvert \\ > 0, & \lvert c_1\rvert < \lvert c_2\rvert \end{cases}$	$\begin{cases} 1 \\ 0 \\ 1 \end{cases}$
